# The long-term benefit of computer-assisted surgical navigation in unicompartmental knee arthroplasty

**DOI:** 10.1186/1749-799X-5-94

**Published:** 2010-12-31

**Authors:** Arpad Konyves, Charles A Willis-Owen, Anthony J Spriggins

**Affiliations:** 1 Sports Surgery and Arthroplasty Fellow, SPORTSMED SA, 32 Payneham Road, Stepney 5069 South Australia; 2 Consultant Orthopaedic Surgeon, SPORTSMED SA, 32 Payneham Road, Stepney 5069 South Australia

## Abstract

We reviewed the outcomes of 30 consecutive primary unicompartmental knee arthroplasties (UKA) performed by a single surgeon for medial compartmental osteoarthritis. Fifteen Allegretto knees were implanted without computer navigation and 15 EIUS knees were implanted with navigation. We compared the survivorship, radiological and clinical outcomes of the two groups at an average of 8.9 years and 6.9 years respectively. The patients were assessed clinically using the Oxford Knee Score (OKS) and radiologically using long-leg weightbearing films and non-weightbearing computed tomography alignment measurements. The overall survivorship was 86.7% at 9 years. A higher proportion of navigated knees were well aligned with a more reproducible position and malaligned knees tended to have a less favourable OKS. However, we found no statistically significant difference in survivorship, clinical outcome and radiological alignment between the two groups.

## Introduction

Unicompartmental knee arthroplasty (UKA) has proved to be a popular option in the treatment of isolated medial compartmental osteoarthritis (OA) with good long term results [1-3]. Isolated medial compartmental OA has been reported to be present in around 21% in males and 12% in females [4] or in 85% of knees with clinical OA [5]. There is little debate that when compared with total knee arthroplasty (TKA), UKA is less invasive, causes less morbidity, better reproduces kinematics, and therefore offers quicker recovery, better range of movement [6] and more physiologic function [7]. However the use of unicompartmental knee replacement has been decreasing in recent years [8,9] and this may be due to the higher overall revision rates compared with TKA in national joint registries. However, revision rates are still acceptable considering the theoretical conservative nature of UKAs and that revision surgery is offered much more readily when compared with TKAs [10]. On the other hand, UKAs skilfully implanted into appropriately selected patients can outperform TKAs over the longer term [11].

Technically UKAs are less forgiving than TKAs and certain considerations must be fulfilled; most importantly overcorrection of the mechanical axis should be avoided [12]. The advent of minimally invasive implantation, which is now the preferred approach with advocators of UKAs, has further increased the difficulty in accurate implantation [13]. Several recent studies have suggested that the radiological position of implants and post-operative limb alignment in UKA is superior following the use of computer navigation [14-18]. Clinical outcome data for computer navigated UKAs is limited, with one study [19] demonstrating no significant differences between function parameters of navigated and non-navigated groups at 2 years. To the best of our knowledge, no published studies have examined mid- to long-term benefits of computer navigation in UKA. This study set out to determine whether more accurate implantation using computer navigation resulted in better mid- to long term survivorship and clinical outcomes.

## Patients and Methods

Between May 2001 and August 2003, 30 consecutive primary medial UKAs were performed in 28 patients by the senior author (AJS). Of these, 15 had a non-navigated Allegretto (Sulzer, Wintherthur, Switzerland) UKA and 15 had a navigated EIUS (Stryker-Howmedica, Allendale, NJ) UKA. These knees had been previously reviewed at a mean of 8 months and 17 months follow-up for another study [17]. The same patients were once again assessed clinically and radiologically for the purposes of this study. We used the same radiological methods and statistical analyses; clinical results had not been examined in the previous study.

**Figure 1:**
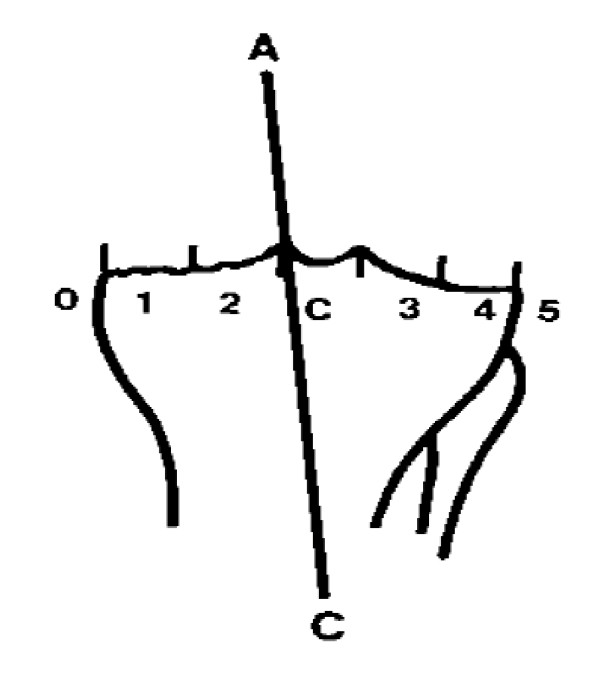
**Kennedy's zones (A-C: mechanical axis)**. After Kennedy and White.

Radiologic examination consisted of weightbearing long leg antero-posterior alignment views as well as CT alignment views as per the Perth protocol. The Perth protocol [20] uses multiple 3-mm-slice images from the hip to the talus to produce coronal, sagittal and axial measurements. Both sets of images were assessed by the same radiologist as the previous study, who was blinded to the treatment method. The zone of the tibial plateau through which the mechanical axis traversed was analysed using the methods described by Kennedy and White [12] (Figure 1). Patients were sent Oxford Knee Scores (OKS) questionnaires and any knee symptoms and range of movement were recorded at clinic reviews. Statistical analysis was performed using Fisher exact test for 2-group comparison, Kaplan-Meier survivor analysis to describe survivorship and logrank tests for 2-group survivorship comparison. Microsoft Excel (Microsoft, Redmond, Washington) and MedCalc statistical software (MedCalc Software bvba, Mariakerke, Belgium) were used for the analysis.

## Results

Of the original 30 knees 3 patients had been revised to total knee replacements, one had been lost to follow-up (counted as a failure in our survivorship analysis), and two patients decided not to participate in the study (both were contacted by phone to ensure that the original UKA was still in situ - and were therefore counted as successes in the survivorship analysis). Twenty-two patients (24 knees) returned the questionnaire and 21 patients (23 knees) attended a radiologic and clinical review. Of the 24 knees 10 had been navigated (9 reviewed) and 14 non-navigated (14 reviewed). Average age at the time of the operation was 59 years (range 41-78) in the navigated group and 61 years (range 44-71) in the non-navigated group. There was no statistical difference in age between the two groups. Average follow-up time was 6.9 years (range 6.4 to 7.4 years) for the navigated group and 8.9 years (range 7.6 to 10.2 years) for the non-navigated group.

### Survivorship

**Figure 2:**
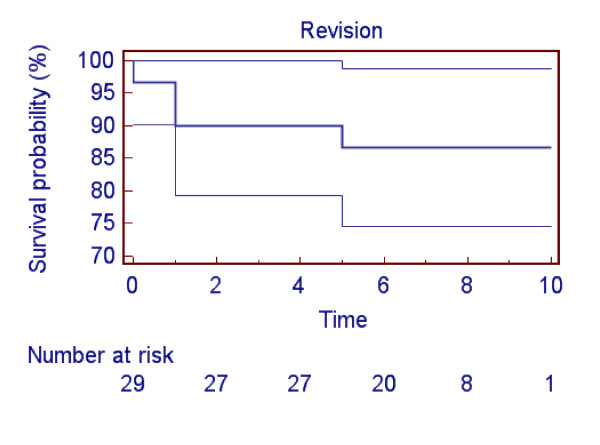
**Kaplan-Meier survival curve of the whole cohort (navigated and non-navigated knees) showing 86.7% survival at 8 years with 95% confidence interval (CI) and the number of knees at risk at the beginning of each year**.

Of the original 28 patients (30 knees), 3 patients (3 knees) had been revised to total knee replacements; all 3 were in the navigated group. Two knees had been revised after one year because of continuing pain and one knee after 5 years because of disease progression. Cumulative survival after 8 years was 86.7% (Figure 2).

Comparison of survival curves between the navigated and non-navigated groups (78.6% vs. 100%) using logrank test showed the difference was not statistically significant (p = 0.0625).

### Radiology

Weightbearing mechanical axis views and CT axis measurements correlated well (r = 0.908) with 4 disagreements and 19 agreements. The disagreements were in the adjacent zones and may represent the effects of weightbearing.

The mechanical axis crossed the tibial plateau at a mean or 42.63% of the tibial width (range 3.33% to 77.50%) with a SD of 19.75%. There was no significant difference between the means of navigated and non-navigated knees (42.4% v. 42.8%; p = 0.96). However there was a higher variance in the non-navigated group, with a SD 22.5% in the non-navigated group versus 15.1% in the navigated group.

Examining Kennedy zones, 16 knees were well aligned (in zone 2 and zone C) (table 1). A higher proportion of navigated knees were well aligned (77% v. 64%), however this difference was not significant using a Fisher exact test.

**Table 1:** Number of patients in Kennedy zones on alignment views.

	Navigated	Non-navigated	Total
Zone 1	1	2	3
Zone 2	2	3	5
Zone C	5	6	11
Zone 3	1	3	4
Zone 4	0	0	0

Comparing the Kennedy zones in our previous and present study, we found that the measurements matched in 10 knees, differing in 13 cases. Of the 13 mismatches the most recent measurements were in adjacent zones in 9 cases. These may have represented an error in measurement or minimal lateral or medial compartmental deterioration. Four knees showed a measurement difference of 2 zones, two of these had severe lateral compartmental degeneration and two had subsidence of the tibial components. One of these had been navigated and one non-navigated.

### Clinical outcome

**Figure 3:**
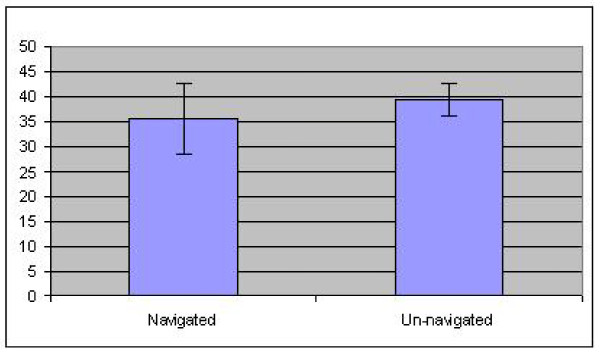
**Graph showing OKS of mean 35.5 (95% CI 28.4 to 42.6) for navigated knees and mean 39.4 (95% CI 35.8 to 42.9) for non-navigated knees (p = 0.35)**.

Eighteen out of 24 knees had continued to do well with good to excellent scores on the OKS. The median OKS was 40 (12 worst, 48 best) with a mean of 37.7 (SD 9). There was no significant difference in scores between the navigated and un-navigated groups (Figure 3).

Although a larger proportion of malaligned knees had a poor to fair OKS than well aligned knees (28% v. 13%), we found no statistically significant difference using the Fisher exact test. This may have been due to the small number of patients in our study.

All but two knees had a range of flexion beyond 100 degrees; we did not find a correlation between range of movement and alignment of the leg. There was one patient in each group with a range of flexion less than 100 degrees, one of whom (non-navigated) reported excellent results (OKS 45) and one (navigated) who reported poor results (OKS 18). Pre-operative movement was not recorded in every case and therefore we did not attempt to make comparisons.

## Discussion

Unicompartmental knee arthroplasty (UKA) is an attractive option for isolated medial compartmental osteoarthritis with good long-term results [1-3]. A substantial proportion of patients undergoing knee arthroplasty are suitable for UKA, which would result in a functionally superior outcome with function similar to the native knee at a reduced cost to the health service [21]. However the use of UKA has been declining [9] in recent years and this may be due to technically challenging surgery and difficulties in the accurate placement of the implants, which is key to a successful clinical outcome.

Computer-assisted surgical navigation has the potential to improve the accuracy of implant positioning, however its effect on clinical outcome is still debatable. The relatively recent introduction of computer navigation means that long-term studies are not available yet. However, short- to mid-term studies in TKAs [22,23] and a short-term study in UKA [19] found no statistical difference between navigated and non-navigated knees.

This study did not demonstrate a significant difference in the longer term survivorship and clinical outcomes of navigated and non-navigated UKAs. A larger proportion of well aligned knees had good or excellent clinical outcomes and a higher proportion of navigated knees were well aligned, though these trends were not statistically significant. The importance of accurate mechanical alignment in TKAs has been debated recently [24] and our poorer (although statistically not significant) survivorship results show that more accurate and reproducible implant positioning may not necessarily lead to a better survival.

Our previous study [17] showed that computer navigation facilitated a higher rate of knees to be in the desired zone for leg alignment. In the present study there is a tendency, but the difference is statistically not significant using the same statistical tests. We demonstrated minor changes in leg alignment over time in 9 knees and substantial changes in 4 knees. It is not clear how much these minor changes represent an actual deterioration and how much they represent an intra-observer error, as only measurements were available from the previous study.

The limitations of our study lie mainly in the small sample size and thus a loss of statistical power. The differences in survival between the two groups was statistically not significant (p = 0.0625), however with longer follow-up this may become significant in favour of the non-navigated group. The implants used in the two groups were different, however both were fully cemented, fixed bearing unicompartmental knees with a similar design rationale. We had good results with the Allegretto, but a change to the EIUS was necessary to enable us to use the navigation system in our hospital. The cohort in our study also represents the initial part of the senior surgeon's learning curve with computer navigation, which may have affected our results unfavourably [25]. At the time of the change the EIUS was relatively new without long-term registry data. The latest National Joint Registry [9] reports higher revision rates for the EIUS (3.3 vs. 1.8 revisions per 100 obs. years) which may be a factor in our survival analysis. Since our navigated cohort followed on our non-navigated group, ranges of follow-up do not overlap. Therefore outcome measures are obtained on average 2 years apart and any difference in the groups may be attributed to a natural disease progression.

Although there is evidence that increased operating times can result in higher infection rates [26], it is our impression that the time spent on setting up the computer referencing does not significantly add to the overall operating time and may even be offset by the time taken to place jigs and perform bone resections.

## Conclusion

This study demonstrates that there is no difference in survivorship and radiological alignment or OKS between navigated and non-navigated UKAs at an average of 6.9 years and 8.9 years, respectively. Long-term follow-up with larger patient groups will be required to establish whether component alignment is a predictor for a successful clinical outcome and to justify the routine use of computer navigation in UKAs.

## Competing interests

The authors declare that they have no competing interests.

## Authors' contributions

AK collected and analysed data and drafted the manuscript; CWO contributed to statistical analysis and revisions of the manuscript; AJS conceived of the study. All authors read and approved the final manuscript.
